# NLRX1 Negatively Regulates Group A *Streptococcus* Invasion and Autophagy Induction by Interacting With the Beclin 1–UVRAG Complex

**DOI:** 10.3389/fcimb.2018.00403

**Published:** 2018-11-14

**Authors:** Chihiro Aikawa, Shintaro Nakajima, Miho Karimine, Takashi Nozawa, Atsuko Minowa-Nozawa, Hirotaka Toh, Shunsuke Yamada, Ichiro Nakagawa

**Affiliations:** ^1^Department of Microbiology, Graduate School of Medicine, Kyoto University, Kyoto, Japan; ^2^Department of Life Science Dentistry, The Nippon Dental University, Tokyo, Japan

**Keywords:** NLRX1, Beclin 1, UVRAG, bacterial invasion, autophagy, Group A *Streptococcus*

## Abstract

Group A *Streptococcus* (GAS) can invade epithelial cells; however, these bacteria are targeted and eventually destroyed by autophagy. Members of the Nod-like receptor (NLR) family are thought to be critical for the autophagic response to invasive bacteria. However, the intracellular sensors within host cells that are responsible for bacterial invasion and the induction of autophagy are largely unknown. Thus, our aim was to examine the role of one such NLR, namely NLRX1, in invasion and autophagy during GAS infection. We found that GAS invasion was markedly increased in NLRX1 knockout cells. This led to the potentiation of autophagic processes such as autophagosome and autolysosome formation. NLRX1 was found to interact with Beclin 1 and UVRAG, members of Beclin1 complex, and knockout of these proteins inhibited invasion and autophagy upon GAS infection. Especially, NLRX1 interacted with Beclin 1 via its NACHT domain and this interaction was responsible for the NLRX1-mediated inhibition of invasion and autophagic processes including autophagosome and autolysosome formation during GAS infection. These findings demonstrate that NLRX1 functions as a negative regulator to inactivate the Beclin 1–UVRAG complex, which regulates invasion and autophagy during GAS infection. Thus, our study expands our knowledge of the role of NLRX1 during bacterial invasion and autophagy and could lead to further investigations to understand pathogen–host cell interactions, facilitating novel targeted therapeutics.

## Introduction

Group A *Streptococcus* (GAS; *Streptococcus pyogenes*), a clinically important pathogen, causes localized respiratory tract and skin infections, as well as, severe human diseases such as toxic shock-like syndrome (Cunningham, 2000). GAS can invade epithelial cells via endocytosis and escape into the cytoplasm by inserting streptolysin O (SLO), a cholesterol-dependent pore-forming cytolysin, into the endosomal membrane (Nakagawa et al., 2004).

Autophagy, a highly conserved biological process that occurs in eukaryotic cells, plays a crucial role in quality and quantity control of the cytoplasm (Mizushima et al., 2008). Accumulating evidence suggests that autophagy is an important innate defense system for non-phagocytic cells, functioning to protect against intracellular pathogens (Levine et al., 2011; Kimmey and Stallings, 2016). During the autophagic process, intracellular GAS bacteria are rapidly sequestered into autophagosomes that are associated with microtubule-associated protein 1 light chain 3 (LC3), resulting in its degradation in the vacuole after fusion with lysosomes (Nakagawa et al., 2004).

Beclin 1 (Atg6/Vps30 in yeast) is known as a key initiator of the autophagic process, and this protein associates with class III type phosphatidylinositol 3-kinase (PI3KC3)/VPS34, leading to the production of phosphatidylinositol 3-phosphate (PtdIns(3)P), thereby modulating membrane dynamics that accompany autophagy (Lamb et al., 2013). Beclin 1 associates with several regulator proteins in addition to PI3KC3/VPS34, and these interaction partners have distinct roles in regulating the autophagic process. For example, the interaction between Beclin 1 and the activating molecule in Beclin 1-regulated autophagy (AMBRA1), UV radiation resistance-associated gene protein (UVRAG), and ATG14 promotes the formation of a Beclin 1–PI3KC3/VPS34 complex, which positively regulates autophagy in mammals (Liang et al., 2006; Fimia et al., 2007; Di Bartolomeo et al., 2010). In contrast, Beclin 1 associates with RUN domain and cysteine-rich domain containing Beclin 1-interacting protein (Rubicon), Bcl-2, and Bcl-xL, which inhibits Beclin 1-induced autophagy (Pattingre et al., 2005; Maiuri et al., 2007; Matsunaga et al., 2009; Zhong et al., 2009).

The innate immune system, which includes autophagy, serves as the first line of defense against invading pathogens and comprises the recognition step for intracellular pathogens. This process is facilitated by several pattern-recognition receptors that sense highly conserved pathogen-associated molecular patterns (PAMPs) (Akira et al., 2006). Regarding these receptors that recognize such PAMPs, NLRs (nucleotide-binding domain leucine-rich repeat containing receptors; NOD-like receptors) facilitate bacterial pathogen recognition and the induction of autophagy inside host cells (Homer et al., 2010; Travassos et al., 2010; Jounai et al., 2011; Irving et al., 2014). For example, it was previously found that NOD1 and NOD2 recognition receptors interact with the autophagy factor ATG16L1, recruiting it to the plasma membrane, and resulting in the enhanced incorporation of *Shigella flexneri* and *Salmonella typhimurium* into autophagosomes (Travassos et al., 2010). In addition, some NLRs such as NLRC4 and NLRP4 were shown to associate with Beclin 1, which in turn negatively regulates autophagy during bacterial infection (Jounai et al., 2011). However, the involvement of the NLRX1–Beclin 1 complex in autophagy in response to bacterial infection remains unknown.

In this study, we examined the role of NLRX1 in invasion and autophagy during GAS infection, and showed that NLRX1 inhibits endocytosis-mediated invasion of GAS bacteria into host epithelial cells, which consequently results in the suppression of autophagy to clear cytoplasmic GAS. Notably, these inhibitory effects on invasion and autophagy were attributed to the interaction between NLRX1 and the Beclin 1–UVRAG complex.

## Materials and methods

### Cell culture and transfection

HeLa cells were purchased from the American Type Culture Collection and cultured in Dulbecco's modified Eagle's medium (DMEM; Nacalai Tesque) supplemented with 10% fetal bovine serum (Gibco) and 50 μg/mL gentamicin (Nacalai Tesque) in a 5% CO_2_ incubator at 37°C. Plasmid transfections were performed using polyethylenimine (Polysciences, Inc.), Lipofectamine 3000 (Invitrogen), or Lipofectamine RNAiMAX (Invitrogen), according to the manufacturers' protocols.

### Group A *streptococcus* strain

Group A *Streptococcus* (GAS) strain JRS4 (M6^+^ F1^+^) was grown in Todd–Hewitt broth (BD Diagnostic Systems, Sparks, MD) supplemented with 0.2% yeast extract (THY), as described previously (Nakagawa et al., 2004).

### Plasmid construction

Gateway cloning technology (Invitrogen) was used to create the vectors indicated as follows. Human *NLRX1* (GenBank Accession No. NM_024618.3), *VPS34* (GenBank Accession No. NM_002647.3), *beclin 1* (GenBank Accession No. NM_003766.4), ATG14 (GenBank Accession No. NM_014924.4), and *UVRAG* (GenBank Accession No. NM_003369.3) were PCR-amplified from human cDNA libraries using the following primer pairs: NLRX1_F: 5′-CACC ATGAGGTGGGGCCACCATTTGCCCAGGGCC-3′ and NLRX1_R: 5′-GCTTCCAGAGCTTCCCAGCTGCTCCAGGAGGG-3′; VPS34_F: 5′-CACCATGGGGGAAGCAGAGAAGTT-3′ and VPS34_R: 5′- TCATTTTCTCCAGTACTGGGC-3′; Beclin 1_F: 5′-CACCATGGAAGGGTCTAAGACGTCCAACAACAGC-3′ and Beclin 1_R: 5′-TCATTTGTTATAAAATTGTGAGGACACCCA-3′; ATG14_F: 5′- CACCATGGCGTCTCCCAGTGGGAAGGGAGCCCGG-3′ and ATG14_R: 5′- TTAACGGTGTCCAGTGTAAGCTTTAAACCA-3′; UVRAG_F: 5′-CACCATGAGCGCCTCCGCGTCGGTCGGGGGCCCC-3′ and UVRAG_R: 5′-TCACTTATCGGAACTCCTGCGCGGCCGGCG-3′. These PCR products were cloned into the pENTR/D-TOPO vector using the pENTR Directional TOPO Cloning Kit (Invitrogen). The entry vector encoding NLRX1 was subcloned into p3xFLAG-C-DEST and pcDNA6.2/C-EmGFP-DEST vectors. The entry vectors encoding VPS34, Beclin 1, ATG14, and UVRAG were subcloned into p3xFLAG-N-DEST and pcDNA6.2/N-EmGFP-DEST vectors.

### Antibodies and other reagents

The following antibodies were used: mouse monoclonal anti-NLRX1 (1:1000; 04–146; Millipore), rabbit polyclonal anti-VPS34/PI3KC3 (38–2100; Invitrogen), rabbit polyclonal anti-Beclin 1 (1:1000; 3738; Cell Signaling Technology), rabbit polyclonal anti-ATG14 (1:1000; A6358; Sigma-Aldrich), rabbit polyclonal anti-UVRAG (1:1000; NBP1-18885; Novus Biologicals), rabbit polyclonal anti-Rubicon (1:1000; ab92388; Abcam), mouse monoclonal anti-LAMP1 (1:100; H4A3; Santa Cruz Biotechnology, Santa Cruz, CA), mouse anti-polyubiquitin (FK2) (1:100; Nippon Bio-Test Laboratories), rabbit monoclonal anti-α-tubulin (1:1000; T6199; Sigma-Aldrich), mouse monoclonal anti-FLAG M2 (1:1000; F1804; Sigma-Aldrich), and mouse monoclonal anti-GFP (1:1000; GF200; 04363-24; Nacalai Tesque). Horseradish peroxidase-conjugated anti-mouse or anti-rabbit IgG (1:5000; Jackson ImmunoResearch Laboratories) were used as secondary antibodies for immunoblotting. The fluorescent secondary antibodies Alexa Fluor 488-conjugated goat anti-rabbit IgG (1:200; Molecular Probes/Invitrogen), or Alexa Fluor 568-conjugated goat anti-mouse IgG (1:200; Molecular Probes/Invitrogen) were used for immunofluorescence.

### Bacterial infection

GAS infections were performed as described previously (Nakagawa et al., 2004). Bacteria grown through mid-log phase were added to cell cultures at a multiplicity of infection of 100, without antibiotics. After 0.5 or 1 h, the infected cells were washed with phosphate-buffered saline (PBS), and then 10% DMEM/FBS with antibiotics (100 μg/mL gentamicin) was added for an appropriate period to eliminate extracellular bacteria. The cells were further cultured for the indicated times.

### Fluorescence microscopy

For immunostaining experiments, cells were washed with PBS, fixed with 4% paraformaldehyde in PBS for 15 min, permeabilized with 0.1% triton X-100 in PBS for 5 min, and then incubated in skim milk blocking buffer (5% skim milk, 2.5% goat serum, 2.5% donkey serum, and 0.02% sodium azide in PBS containing 0.1% gelatin) or BSA blocking solution (2% BSA and 0.02% sodium azide in PBS) at room temperature for 1 h. Subsequently, cells were incubated with primary antibodies diluted with blocking solution at 4°C overnight, washed with PBS, and then probed with secondary antibodies. Bacterial and cellular DNA was stained with 4′,6-diamidino-2-phenylindole (DAPI; Dojindo) in PBS. For quantification of GAS invasion, HeLa cells were seeded on coverslips, infected with GAS, and fixed as described. Extracellular GAS bacteria were first stained with Alexa Fluor-488-conjugated rabbit polyclonal anti-GAS antibody, followed by permeabilization and staining of intracellular GAS with Alexa-Fluor-568-conjugated rabbit polyclonal anti-GAS antibody (1:200; PAB13831; Abnova). GAS invasion was calculated by dividing the number of Alexa-Fluor-568-stained GAS bacteria by the total number of GAS bacteria (Alexa Fluor-488 and Alexa Fluor-568 stained). At least 300 GAS bacteria were scored from three independent experiments. Fluorescence confocal microscopy images were acquired using an FV1000 laser-scanning microscope (Olympus).

### Measurement of GcAV formation efficiency

To quantify GcAV-formation efficiency, fluorescence microscopy samples were prepared as described previously herein, and were examined using confocal microscopy. The rate of GcAV formation was expressed as the ratio of GcAV-harboring cells to GAS-infected cells. More than 500 cells were analyzed for each assay.

### Bacterial invasion assays

Cells were cultured in 24-well culture plates and infected as described. After an appropriate incubation time, the infected cells were washed with PBS and lysed in distilled water. Serial dilutions of lysates were plated on THY agar (3% Todd Hewitt Broth, 0.2% yeast extract, and 1.5% agar) plates, and colonies were counted. Data are presented as the ratio of total intracellular GAS bacteria at 2 h post-infection to total attached GAS bacteria at 0.5 or 1 h post-infection.

### Generation of knockout (KO) cell lines using CRISPR/Cas9

The CRISPR/Cas9 system (Mali et al., 2013) was used to knockout NLRX1, Beclin 1, or UVRAG as described previously (Oda et al., 2016). We selected the CRISPR guide RNA (gRNAs) that targeted an exon common to all splicing variants of the gene of interest (target sequence: NLRX1, 5′-GAGCTGTGCTAGCTCAGCT-3′, Beclin 1, 5′-TCCAACAACAGCACCATGC-3′, UVRAG, 5′-GAGATGAGCGCCTCCGCGT-3′, Rubicon, 5′-CAGTTCAGCTCACGTGATT-3′). For CRISPR/Cas9 gene editing, HeLa cells were transfected with a gRNA-hyg vector containing the CRISPR target sequence and hCAS9 vector (41815; Addgene). Two days after transfection, the untransfected cells were removed by selection with 300 μg/mL hygromycin B (Nacalai Tesque) and 750 μg/mL geneticin (G418; Nacalai Tesque). Single colonies were expanded in 24-well plates, and depletion of the target gene was confirmed by immunoblotting. As a secondary screen for KO lines, genomic DNA was isolated from cells and the genomic regions were amplified by PCR. These PCR products were sequenced to confirm the presence of the desired frameshift insertions and deletions.

### RNA interference

Stealth RNAi oligonucleotides were used for siRNA experiments (Invitrogen). The sequences used were as follows: human Atg14-siRNA sense, 5′-GGCAAAUCUUCGACGAUCCCAUAUA-3′ and antisense, 5′-UAUAUGGGAUCGUCGAAGAUUUGCC-3′; human Vps34-siRNA sense, 5′-CCCAUGAGAUGUACUUGAACGUAAU-3′ and antisense, 5′-AUUACGUUCAAGUACAUCUCAUGGG-3′. As a negative control, Medium GC duplex of stealth RNAi Negative Control Duplexes (Invitrogen) was used. The Stealth RNAi oligonucleotides were transfected into HeLa cells using Lipofectamine RNAi MAX according to the manufacturer's protocols.

### Immunoprecipitation

Cells were harvested, washed with PBS, and lysed in a lysis buffer containing 50 mM tris-HCl pH 7.5, 150 mM NaCl, 100 mM NaF, 10 mM EGTA, 1 mM Na_3_VO_4_, 5 μM ZnCl_2_, 10% glycerol, 1% triton X-100, and a proteinase inhibitor cocktail (Nacalai Tesque) for 30 min at 4°C. The lysates were then centrifuged, and the obtained supernatants were pre-cleared by incubating them with Protein G Sepharose 4B (GE Healthcare Life Sciences) for 1 h at 4°C. After brief centrifugation, the supernatants were reacted with anti-GFP antibodies at 4°C overnight, and then Protein G Sepharose beads were added and incubated with rotation for 1 h at 4°C. Immunoprecipitates were collected by brief centrifugation, and the mixtures were washed five times with washing buffer containing 50 mM Tris-HCl (pH 7.5), 150 mM NaCl, and 0.1% triton X-100, and analyzed by immunoblotting.

### Statistical analysis

All data represented are from three individual independent replicates unless otherwise stated. Values are expressed as the mean ± standard deviation (SD). Data were tested by performing a two-tailed Student's *t*-test. *P*-values < 0.05 were considered to indicate statistical significance, and are marked by ^*^ for *P* < 0.05 and ^**^ for *P* < 0.01.

## Results

### NLRX1 suppresses gas invasion into host cells via endocytosis

**Figure 1:**
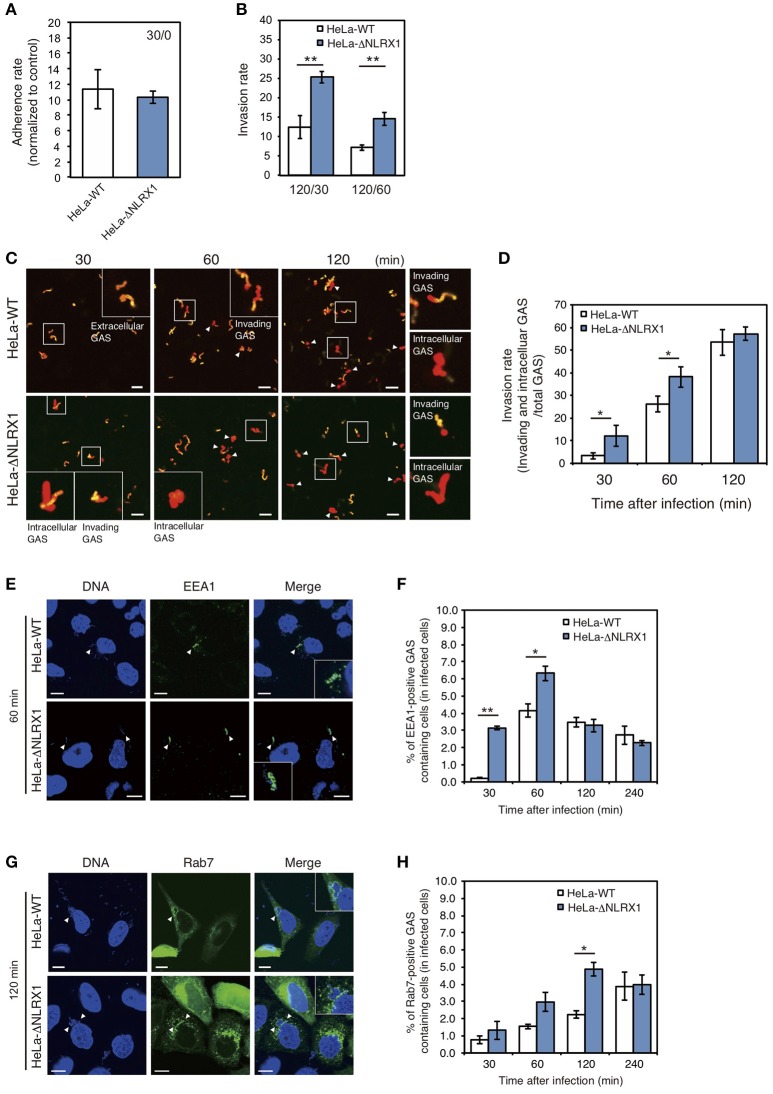
NLRX1 inhibits endocytosis-mediated Group A *Streptococcus* (GAS) invasion. **(A,B)** Adherence **(A)** and invasion **(B)** rates of GAS. HeLa wild-type and NLRX1 KO cells were infected with GAS (MOI = 100). After 30 or 60 min of infection, cells were washed with PBS and further incubated for 60 min with DMEM/10% FCS with gentamicin (100 μg/mL) to kill extracellular bacteria. Cells were disrupted with distilled water and serial dilutions of cellular extracts were plated on THY agar plates; colony counting was then performed. The data presents the invasion rate as the ratio of total intracellular GAS 120 min after infection to total adherent GAS at 30 or 60 min after infection. Data are representative of ≥ three independent experiments. ^**^P < 0.01. **(C)** Representative immunofluorescence image used for quantification of GAS invasion in **(D)**. Extracellular/cell-associated GAS (orange) and intracellular GAS (red) were differentiated by staining with different Alexa-Fluor-conjugated GAS antibodies. GAS stained both orange and red were scored as invading bacteria (white arrow). **(D)** Quantification of GAS invasion into NLRX1 KO cells. Data were obtained by scoring at least 300 bacteria chains. Data are representative of ≥ three independent experiments. ^*^P < 0.05. **(E,G)** Confocal microscopic images of EEA1 **(E)**- or Rab7 **(G)**-positive compartments containing GAS in HeLa wild-type and NLRX1 KO cells. Cells were transfected with EmGFP-Rab7, or immunostained with an anti-EEA1 antibody to visualize each endosomal marker. Cellular and bacterial DNA was stained with DAPI (blue). White arrowheads show EEA1- or Rab7-positive compartments containing GAS. Scale bars, 10 μm. **(F,H)** The number of cells containing EEA1- or Rab7-positive GAS was counted and presented as the percentage of the total number of GAS-infected cells. HeLa wild-type and NLRX1 KO cells were infected with GAS for indicated times. The data shown represent results from > 200 infected cells and include the mean value ± SD from three independent experiments. ^**^P < 0.01, ^*^P < 0.05.

Initially, to investigate the effect of NLRX1 on GAS invasion, we evaluated cell invasion using NLRX1 KO HeLa cells established by genome editing (Supplementary Figure S1). Although the adherence rate of GAS was not different between wild-type and NLRX1 KO cells (Figure 1A), the number of invaded GAS bacteria increased by ~1.5–2 fold in NLRX1 KO cells, compared to that in wild-type cells (Figure 1B). We also examined the invasive ability of GAS in both WT and NLRX1 KO cells by confocal immunofluorescence microscopy (Figure 1C) and observed an increase in invading GAS bacteria in NLRX1 KO cells compared to that in wild-type cells (Figure 1D). To further assess the relationship between NLRX1 and endocytosis-mediated invasion of GAS, we detected the early endosome markers EEA1 and late endosome marker Rab7. At 30 and 60 min after infection, the percentage of EEA1-positive compartments containing GAS in NLRX1 KO cells was significantly increased compared to that in wild-type cells (Figures 1E,F). Similarly, a significant increase in Rab7-positive compartments containing GAS in NLRX1 KO cells was observed, as compared to that in wild-type cells, at 120 min after infection (Figures 1G,H). These results suggest that endogenous NLRX1 inhibits GAS endocytosis-mediated invasion into host cells.

### NLRX1 inhibits the autophagic process during GAS infection

**Figure 2:**
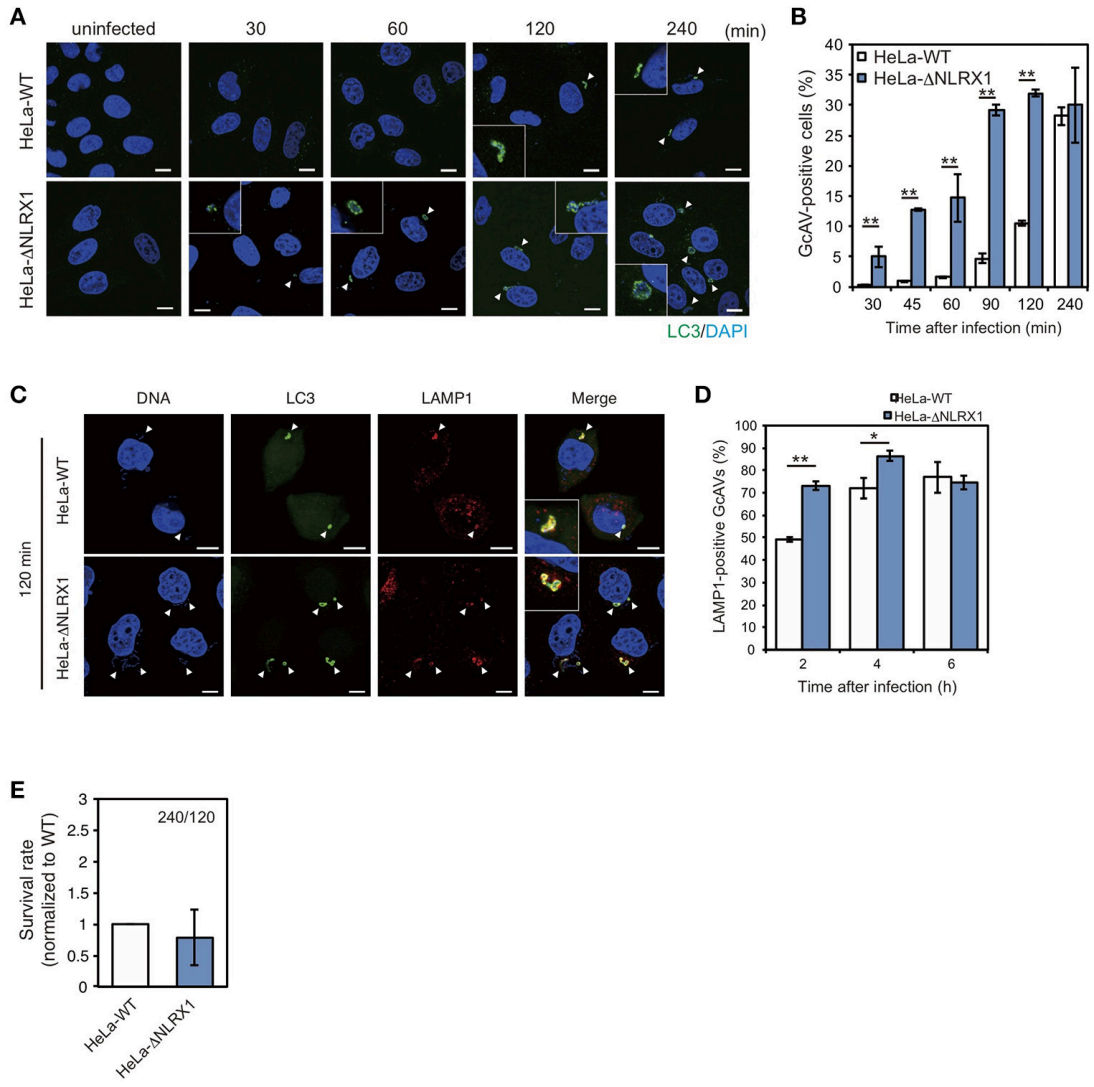
NLRX1 inhibits Group A *Streptococcus* (GAS)-induced autophagy. **(A)** Confocal micrographs of HeLa wild-type cells and NLRX1 KO cells transfected with EmGFP-LC3 and infected with GAS for the indicated times. Cellular and bacterial DNA was stained with DAPI. White arrowheads show GcAV-positive cells. Scale bars, 10 μm. **(B)** Quantification of the number of GcAV-positive HeLa wild-type and NLRX1 KO cells. Data are mean ± SD for 500 GAS-infected cells counted per each experiment (*n* = 3). ^**^P < 0.01. **(C)** Confocal micrographs of HeLa wild-type and NLRX1 KO cells transfected with EmGFP-LC3 and infected with GAS for 120 min. Cells were stained with antibody against LAMP1 and then with Alexa 594-conjugated secondary antibody. Cellular and bacterial DNA was stained with DAPI. White arrowheads show LAMP1-positive GcAVs. Scale bars, 10 μm. **(D)** Co-localization rate of GcAVs with lysosomes in HeLa wild-type and NLRX1 KO cells at the indicated times after infection. Data are mean ± SD of 200 GAS-infected cells counted per each experiment (*n* = 3). ^**^P < 0.01, ^*^P < 0.05. **(E)** Rate of intracellular survival of GAS in HeLa wild-type and NLRX1 KO cells. Each cell type was infected with GAS, and the number of intracellular viable GAS bacteria was determined by colony counting and is presented as the ratio of intracellular live GAS at 240 min to intracellular GAS at 120 min. Data are representative of three independent experiments.

Next, we assessed the effect of NLRX1 on autophagy during GAS infection. Since NLRX1 was suggested to regulate the GAS-related invasion pathway, we assumed that an increased number of invading bacteria in NLRX1 KO cells would affect autophagosome formation. HeLa wild-type cells and NLRX1 KO cells expressing emerald green fluorescent protein-LC3 (EmGFP-LC3) were infected with GAS. As expected, the number of GcAV-positive cells in NLRX1 KO cells was higher during the early stage of infection (30–120 min) compared to that in wild-type cells (Figures 2A,B). After maturation and lysosome fusion, the autophagosome forms a single-layered autolysosome, in which its cellular contents are ultimately degraded (Mizushima et al., 2008). Because the increase in GcAV formation in NLRX1 KO cells occurred during GAS infection, we assumed that autolysosome formation and intracellular GAS survival were also affected in these cells. As expected, autolysosome formation was significantly promoted in NLRX1 KO cells compared to that in HeLa wild-type cells at 120 and 240 min after infection (Figures 2C,D). Although the number of invaded GAS bacteria increased in NLRX1 KO cells, survival rates of these bacteria in NLRX1 KO cells were similar to those in HeLa wild-type cells due to enhanced autolysosome formation (Figure 2E). These results suggest that NLRX1 suppresses GAS-induced autophagy by inhibiting GAS invasion.

### NLRX1 inhibits GAS invasion by interacting with the beclin 1–UVRAG complex

**Figure 3:**
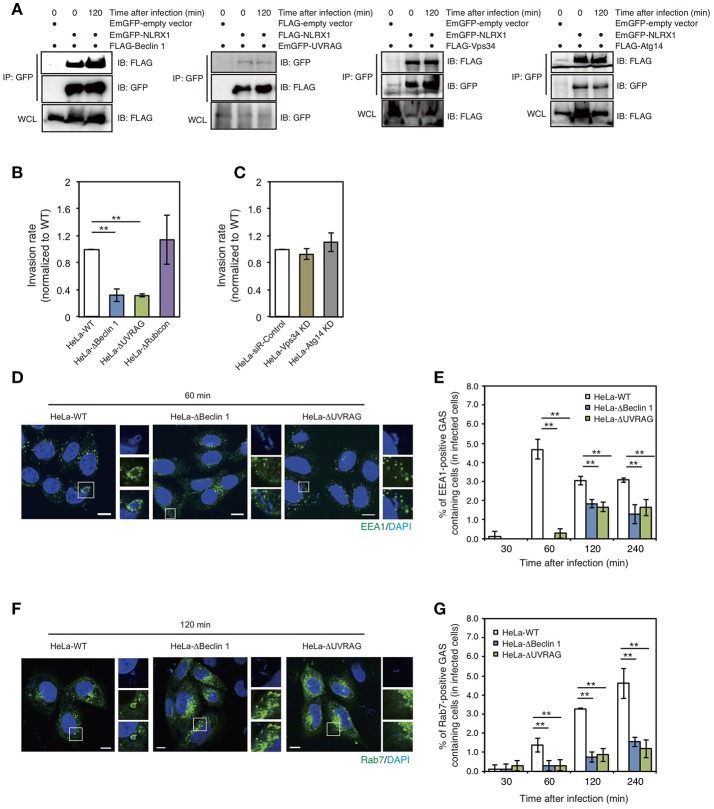
The NLRX1–Beclin 1–UVARAG complex regulates Group A *Streptococcus* (GAS) invasion by endocytosis. **(A)** NLRX1 interacts with the Beclin 1 complex. HeLa cells were transfected with EmGFP-empty vector or EmGFP-tagged Beclin 1 complex proteins (Beclin1, Vps34/PI3KC3, and Atg14) with FLAG-NLRX1. Similarly, cells were transfected with FLAG-empty vector or FLAG-tagged UVARG with EmGFP-NLRX1. Cell lysates were subjected to immunoprecipitations with anti-FLAG or—GFP (for UVRAG) antibody. The immunoprecipitated proteins and total cell lysates were analyzed by immunoblotting with anti-GFP or—FLAG (for UVRAG) antibody. **(B,C)** Invasion rate of GAS in HeLa wild-type, Beclin 1 KO, UVRAG KO, Rubicon KO, Vps34/PI3KC3 KD, and Atg14 KD cells. ^**^P < 0.01. **(D,F)** Confocal microscopic images of EEA1 **(D)**- or Rab7 **(F)**-positive compartments containing GAS in HeLa wild-type, Beclin1, and UVRAG KO cells. Cells were transfected with EmGFP-Rab7, or immunostained with an anti-EEA1 antibody to visualize each endosomal marker. Cellular and bacterial DNA was stained with DAPI (blue). Scale bars, 10 μm. **(E,G)** The number of cells containing EEA1 **(D)**-, or Rab7 **(F)**-positive GAS were counted and presented as the percentage of the total number of GAS-infected cells. HeLa wild-type, Beclin 1, and UVRAG KO cells were infected with GAS for the indicated times. The data shown represent results from > 200 infected cells and indicate the mean value ± SD from three independent experiments. ^**^P < 0.01.

Beclin 1 is one of the most widely characterized proteins involved in the regulation of autophagy. However, accumulating evidence suggests that Beclin 1 is also involved in multiple vesicle trafficking pathways other than autophagy (Wirawan et al., 2012). Our previous study also showed that the interaction between Bcl-xL and the Beclin 1–UVRAG complex regulates GAS invasion (Nakajima et al., 2017). Therefore, we investigated the interaction between NLRX1 and the Beclin 1 complex. Immunoprecipitation assays demonstrated the interaction between NLRX1 and Beclin 1, UVRAG, VPS34/PI3KC3, and ATG14 (Figure 3A). Next, to address the effect of the Beclin 1 complex on NLRX1-mediated inhibition of GAS invasion, the invasive ability of GAS bacteria in Beclin 1, UVRAG, and Rubicon KO cells and ATG14 and VPS34/PI3KC3 knockdown (KD) cells (Supplementary Figure S2) was evaluated. The invasion of GAS in Beclin 1 and UVRAG KO cells was decreased by approximately one third, compared to that in wild-type cells, whereas the number of intracellular bacteria was not altered in Rubicon KO and VPS34 and ATG14 KD cells, compared to that in wild-type or siRNA control cells (Figures 3B,C). Furthermore, the percentage of EEA1- and Rab7-positive compartments containing GAS in Beclin 1 and UVRAG KO cells decreased significantly, compared to that in wild-type cells during infection (Figures 3D–G). These results suggest that NLRX1 inhibits GAS invasion by interacting with the Beclin 1–UVRAG complex.

### NLRX1 inhibits GAS-induced autophagy by interacting with the beclin 1–UVRAG complex

**Figure 4:**
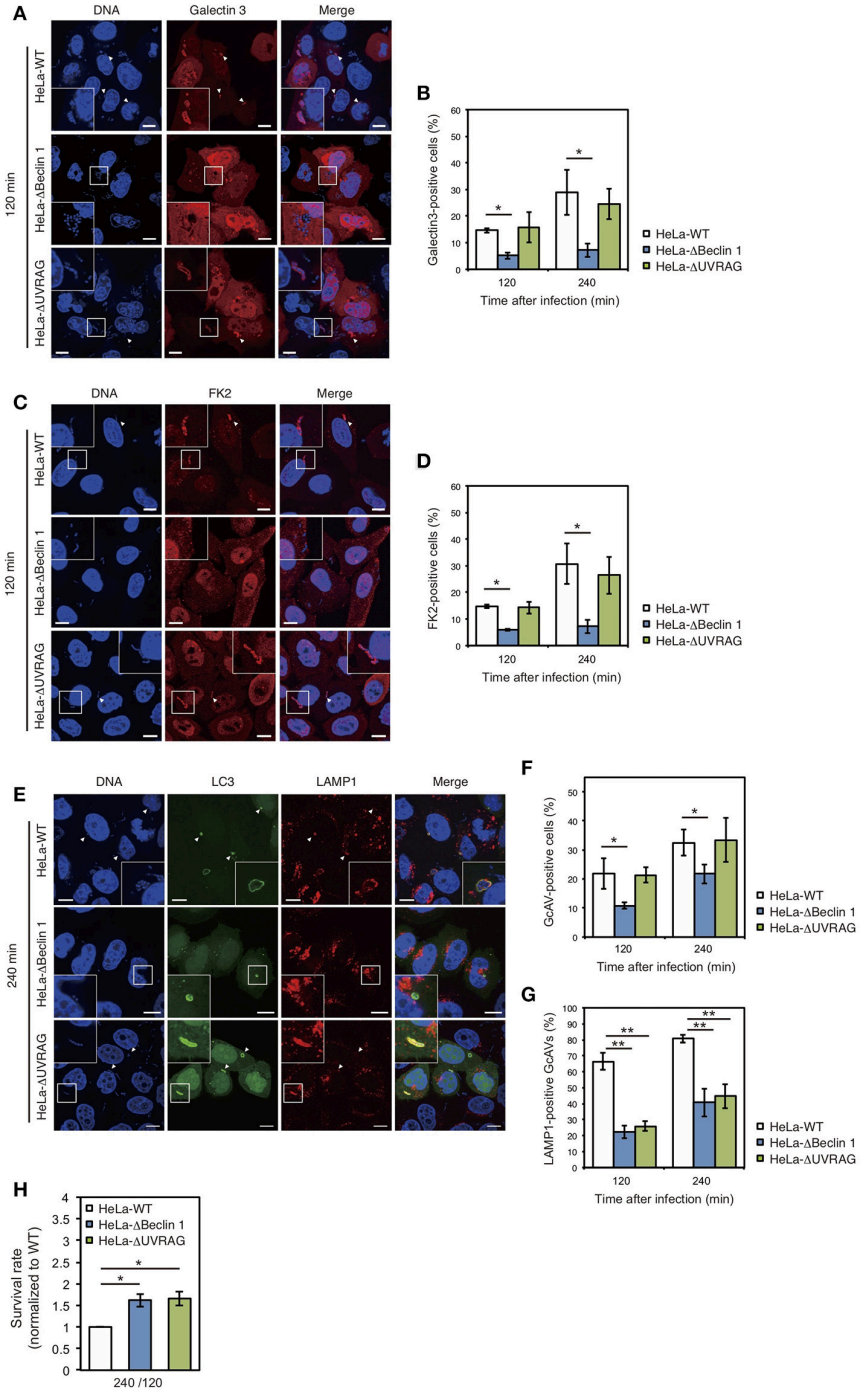
The NLRX1–Beclin 1–UVARAG complex regulates Group A *Streptococcus* (GAS)-induced autophagy. **(A)** HeLa wild-type, Beclin 1, and UVRAG KO cells transfected with mCherry-Galectin3 were infected with GAS for 120 min. Cellular and bacterial DNA was stained with DAPI. White arrowheads show Galectin3-positive cells. Scale bars, 10 μm. **(B)** Quantification of the number of Galectin3-positive HeLa wild-type, Beclin 1, and UVRAG KO cells. Data are mean ± SD for 200 GAS-infected cells counted per each experiment (*n* = 3). ^*^P < 0.05. **(C)** HeLa wild-type, Beclin 1, and UVRAG KO cells were infected with GAS for 120 min and stained with antibody to FK2 and then with Alexa 594-conjugated secondary antibody to visualize ubiquitin-positive GAS. Cellular and bacterial DNA was stained with DAPI. White arrowheads show FK2-positive GAS. Scale bars, 10 μm. **(D)** Quantification of the number of FK2-positive HeLa wild-type, Beclin 1, and UVRAG KO cells. Data are mean ± SD for 200 GAS-infected cells counted per each experiment (*n* = 3). ^*^P < 0.05. **(E)** HeLa wild-type, Beclin 1, and UVRAG KO cells were transfected with EmGFP-LC3 and infected with GAS for 240 min; cells were stained with antibody against LAMP1 and then with Alexa 594-conjugated secondary antibody. Cellular and bacterial DNA was stained with DAPI. White arrowheads show LAMP1-positive GcAVs. Scale bars, 10 μm. **(F)** Quantification of the number of GcAV-positive HeLa wild-type, Beclin 1, and UVRAG KO cells. Data are mean ± SD for 500 GAS-infected cells counted per each experiment (*n* = 3). ^*^P < 0.05. **(G)** Co-localization rate of GcAVs with lysosomes in HeLa wild-type, Beclin 1, and UVRAG KO cells at the indicated times after infection. Data are mean ± SD of 200 GAS-infected cells counted per each experiment (*n* = 3). ^**^P < 0.01. **(H)** The rate of intracellular survival of GAS bacteria in HeLa wild-type, Beclin 1, and UVRAG KO cells. Each cell type was infected with GAS, and the number of intracellular viable GAS bacteria was determined by colony counting and is presented as the ratio of intracellular live GAS bacteria at 240 min to intracellular GAS bacteria at 120 min. Data are representative of three independent experiments.

We also examined the effect of Beclin 1 and UVRAG on GAS-induced autophagy. Since GAS bacteria secrete SLO to disrupt the endosomal membrane (Nakagawa et al., 2004) and ubiquitinated cytoplasmic GAS bacteria are targeted by autophagy (von Muhlinen et al., 2010), we used Galectin-3, a marker of damaged endomembranes (Paz et al., 2010), and a FK2 polyubiquitin antibody. The percentage of Galectin-3-positive GAS in Beclin 1 KO cells was approximately half of that in wild-type cells at 120 and 240 min after infection, whereas there was no difference between wild-type and UVRAG KO cells (Figures 4A,B). Similarly, the percentage of ubiquitinated GAS decreased in Beclin 1 KO, but not in UVRAG KO, cells compared to that in wild-type cells (Figures 4C,D). In addition, the number of GcAV-positive cells was also lower in Beclin 1 KO, but not in UVRAG KO, cells compared to that in HeLa wild-type cells (Figures 4E,F). Notably, the rates of LAMP1-positive GcAV in both Beclin 1 and UVRAG KO cells were lower than those in wild-type cells (Figures 4E,G). Similar to the lower efficiency of autolysosome formation in Beclin 1 and UVRAG KO cells, no significant difference was observed in GAS survival rates between KO and wild-type cells (Figure 4H) despite a decrease in the number of invading GAS bacteria (Figure 3B). These results suggest that although UVRAG involves the invasion process and autolysosome formation by cooperating with Beclin 1, the regulation of invasion and autophagy during NLRX1-mediated GAS infection is due to the interaction between NLRX1 and Beclin 1.

### The NACHT domain of NLRX1 interacts with beclin 1 and inhibits invasion and autophagy during GAS infection

**Figure 5:**
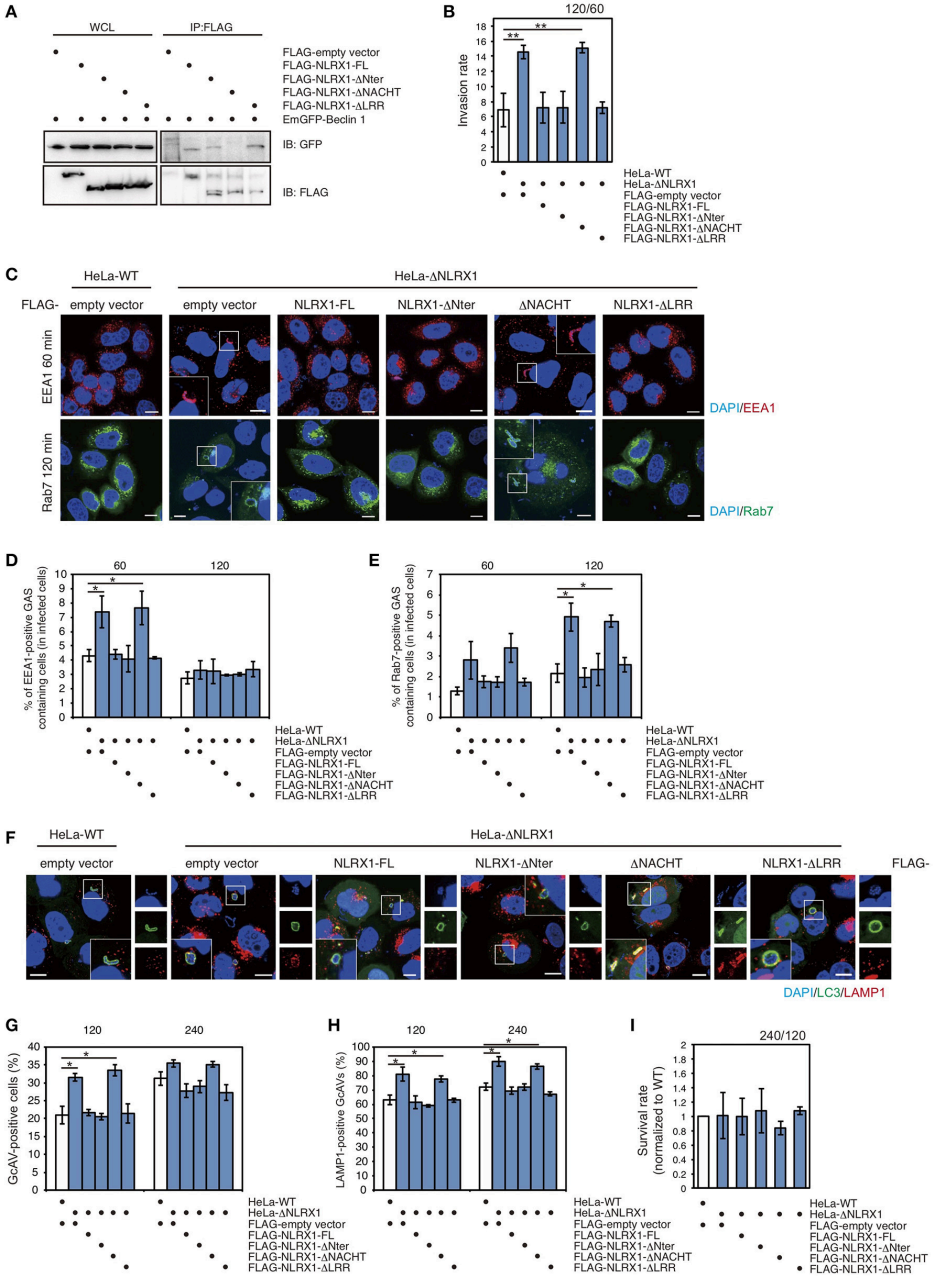
The NACHT domain of NLRX1 interacts with Beclin 1 and is responsible for inhibition of invasion and autophagy during Group A *Streptococcus* (GAS) infection. **(A)** NLRX1 interacts with the Beclin 1 complex through its NACHT domain. HeLa cells were transfected with FLAG-empty vector or FLAG-tagged NLRX1 deletion mutants and EmGFP-Beclin 1, and subjected to immunoprecipitation with an anti-FLAG antibody. The immunoprecipitated proteins and total cell lysates were analyzed by immunoblotting with an anti-GFP antibody. **(B)** Invasion rate of GAS in HeLa wild-type and NLRX1 KO cells transfected with NLRX1 deletion mutants. ^**^P < 0.01. **(C)** Confocal microscopic images of EEA1- or Rab7-positive compartments containing GAS in HeLa wild-type and NLRX1 KO cells transfected with NLRX1 deletion mutants. Cells were transfected with EmGFP-Rab7 or immunostained with an anti-EEA1 antibody. Cellular and bacterial DNA was stained with DAPI. Scale bars, 10 μm. **(D,E)** The number of cells containing EEA1-, or Rab7-positive GAS were counted and presented as the percentage of the total number of GAS-infected cells. HeLa wild-type and NLRX1 KO cells transfected with NLRX1 deletion mutants were infected with GAS. The data shown represent results from > 200 infected cells and indicate the mean value ± SD from three independent experiments. ^*^P < 0.05. **(F)** HeLa wild-type or NLRX1 KO cells transfected with both EmGFP-LC3 and NLRX1 deletion mutants were infected with GAS for 240 min, and stained with an antibody against LAMP1 and then with an Alexa 594-conjugated secondary antibody. Cellular and bacterial DNA was stained with DAPI. Scale bars, 10 μm. **(G)** Quantification of the number of GcAV-positive HeLa wild-type cells and NLRX1 KO cells transfected with NLRX1 deletion mutants. Data are mean ± SD for 500 GAS-infected cells counted per each experiment (*n* = 3). ^*^P < 0.05. **(H)** Colocalization rate of GcAVs with lysosomes in HeLa wild-type and NLRX1 KO cells transfected with NLRX1 deletion mutants. Data are mean ± SD of 200 GAS-infected cells counted per each experiment (n = 3). ^*^P < 0.05. **(I)** The rate of intracellular survival of GAS in HeLa wild-type and NLRX1 KO cells transfected with NLRX1 deletion mutants. The number of intracellular viable GAS bacteria was determined by colony counting and is presented as the ratio of intracellular live GAS bacteria at 240 min to intracellular GAS bacteria at 120 min. Data are representative of three independent experiments.

To identify the domain of NLRX1 responsible for interaction with Beclin 1, a series of deletion mutants was constructed (Supplementary Figure S3). NLRX1 has three unique protein domains: an N-terminal effector domain containing a mitochondria-specific addressing sequence; a central NACHT domain; a C-terminal leucine-rich repeat (LRR) domain. Confocal microscopy showed that all the deletion constructs (ΔNter, ΔNACHT, and ΔLRR) co-localized with the mitochondrial-specific marker MitoTracker, as well as, full-length NLRX1 (NLRX1-FL) (Supplementary Figure S3C). Immunoprecipitation assays showed that ΔNter and ΔLRR interacted with Beclin 1 as well as NLRX1-FL, but that ΔNACHT lost its ability to interact with Beclin1 (Figure 5A), indicating that NLRX1 interacts with Beclin 1 through its NACHT domain.

Finally, to confirm whether the NACHT domain of NLRX1 functions to inhibit GAS invasion and the induction of autophagy, we examined cell-invasion ability of GAS and autophagic responses in NLRX1 KO HeLa cells transfected with a series of NLRX1 deletion mutants. An increased number of invading GAS bacteria and percentage of EEA1- and Rab7-positive compartments containing GAS in NLRX1 KO cells were reduced to the same level observed in wild-type cells after transfection with NLRX1-FL, ΔNter, and ΔLRR, whereas transfection of ΔNACHT retained the enhanced GAS invasion (Figures 5B–E). We further examined the efficiency of GcAV and autolysosome formation. The rates of GcAV- and autolysosome-positive cells in NLRX1-FL-, ΔNter-, and ΔLRR-overexpressing NLRX1 KO cells decreased to almost the same rate observed in wild-type cells, whereas transfection of ΔNACHT retained the enhancing effect on GcAV and autolysosome formation, compared to that observed in NLRX1 KO cells (Figures 5F–H). No significant differences in GAS survival rates were observed between HeLa wild-type and NLRX1 KO cells transfected with the NLRXI deletion mutants (Figure 5I). These results clearly indicate that NLRX1 interacts with Beclin 1 via its NACHT domain, which results in the inhibition of endocytosis-mediated invasion and autophagy during GAS infection.

## Discussion

Invasion into host cells provides an intracellular niche, which not only protects GAS bacteria from the primary extracellular defense systems and/or antibiotics but also facilitates to colonization and dissemination. Whereas the beneficial aspects of endocytosis-mediated GAS invasion have been shown, host cells have multiple highly developed elimination systems to protect against invading pathogens, such as autophagy, bactericidal ROS generation, and apoptotic cell death for removing infected cells by programmed suicide (Nakagawa et al., 2001, 2004; Aikawa et al., 2010).

NLRs act as pattern recognition receptors for invading pathogens and activate/inactivate autophagic signaling via interactions with ATG proteins (Deretic et al., 2013). For example, NLRP4 negatively regulates autophagic processes in response to GAS infection through an interaction with Beclin 1. In addition, recent research has shown that interactions between NLRX1 and mitochondrial translation elongation factor Tu regulate virus-induced autophagy through the association between the latter and Atg5–12 and Atg16L1 (Lei et al., 2012). Although increasing evidence suggests that NLRs participate in the regulation of autophagy, little is known about the precise role of many of these proteins in the autophagic machinery during bacterial infection.

Here, we demonstrated that the interaction between NLRX1 and Beclin 1 and UVRAG inhibits GAS invasion into host epithelial cells by endocytosis, which results in the suppression of autophagic processes. We first showed that NLRX1 regulates GcAV formation and that this is attributed to the number of invading GAS bacteria. Second, NLRX1 was also found to regulate GcAV-lysosome fusion, which consequently affects the intracellular survival of GAS. Overall, these results suggest that NLRX1 inhibits GAS-induced autophagy by suppressing GAS invasion. Therefore, we focused on members of the Beclin 1 complex because this complex has been shown to mediate multiple vesicle trafficking events including endocytosis and vacuolar protein sorting, in addition to its crucial role in regulating autophagy (Kihara et al., 2001; Itakura et al., 2008; Liang et al., 2008; Funderburk et al., 2010). Our data revealed that NLRX1 interacts with Beclin 1 and several components of the Beclin 1 complex and that the function of NLRX1 in GAS invasion depends on its interaction with Beclin 1 and UVRAG, but not with VPS34/PI3KC3, ATG14, and Rubicon. In many cases, activation of PI3K is required for GAS host cell invasion and the induction of autophagy (Ozeri et al., 2001; Purushothaman et al., 2003; Wang et al., 2007; Lamb et al., 2013). Indeed, an increased number of invading GAS bacteria and GcAVs were observed in NLRX1 KO cells. Although VPS34/PI3KC3, a member of the PI3K complex, was previously shown to be involved in both endocytic and autophagic processes (Juhasz et al., 2008; Jaber et al., 2016), silencing of VPS34/PI3KC3 did not affect GAS, suggesting that VPS34/PI3KC3 does not contribute to this process. This result is supported by previous reports demonstrating that only PtdIns(3,4,5)P_3_, generated by class I PI3K (Vanhaesebroeck et al., 2012), and PtdIns(3,4)P_2_, generated by PI3KC2α (Leibiger et al., 2015), can recruit the kinase AKT, which is involved in actin cytoskeleton rearrangements during GAS invasion.

A previous study revealed that Beclin 1 forms two distinct PI3KC3 complexes with ATG14 and UVRAG and that the ATG14-containing Beclin 1 complex is required for autophagosome formation, whereas the UVRAG-containing Beclin 1 complex is crucial for maturation of the autophagosome and endosome (Itakura et al., 2008). Furthermore, additional binding of Rubicon to the UVRAG–Beclin 1 complex inhibited the maturation of autophagosomes and endosomes (Matsunaga et al., 2009). ATG14 localizes to the endoplasmic reticulum, isolation membrane, and autophagosome (Matsunaga et al., 2009), whereas UVRAG localizes to the endosome and lysosome. Although both ATG14 and UVRAG were shown to be involved in the endocytic pathway, as well as, the regulation of autophagy (Liang et al., 2008; Kim et al., 2012; Pirooz et al., 2014), knockdown of this protein did not affect GAS invasion. Conversely, defective GAS invasion mediated by endocytosis in Beclin 1 KO cells was phenocopied by UVRAG KO cells. Additionally, we previously showed that ATG14 clearly contributes to autophagosome formation under starvation conditions, whereas it is dispensable for autophagosome formation in the presence of GAS (Nakajima et al., 2017). Therefore, Beclin 1 might organize distinct Beclin 1 complexes that depend on cellular environmental changes, and could mainly form the UVRAG-containing complex during GAS infection.

Our results indicate that the effects on autophagy mediated by NLRX1–UVRAG interactions are partially distinct from those mediated by NLRX1–Beclin1 interactions. Beclin 1 and UVRAG appear to act cooperatively in the machinery of GcAV–lysosome fusion because autolysosome formation during GAS infection was inhibited and intracellular survival of GAS was promoted in both KO cells. In contrast, decreased numbers of damaged endomembranes and ubiquitin-positive GAS bacteria, which represent processes that are required for autophagosome targeting, were observed only in Beclin 1 KO cells, and not in UVRAG KO cells. Although the final destination of GAS inside the host seems to be similar (because both Beclin 1 and UVRAG are involved in GcAV-lysosome fusion), these results suggest another role for Beclin 1, different from that of UVRAG, in the transition of GAS bacteria from endosomes to the cytoplasm and in bacterial targeting by autophagosomes. Therefore, further studies are required to elucidate how Beclin 1 regulates these processes during GAS-induced autophagy.

We also showed that the NACHT domain of NLRX1 interacts with Beclin 1 and inhibits GAS invasion and autophagic processes. This result is mostly consistent with evidence showing that the NACHT domains of NLRs including NLRC4, NLRP3, NLRP4, and NLRP10 can interact with Beclin 1 and that the interaction between the NACHT domain of NLRP4 and Beclin 1 inhibits GcAV formation and the maturation of autophagosomes to autolysosomes (Jounai et al., 2011). Of note, we provide additional evidence showing that the inhibition of GcAV formation was due not only to the inactivation of endocytosis (i.e., the lower number of damaged-endomembranes and ubiquitin-positive GAS bacteria), but also to a decrease in the number of invading GAS bacteria.

There are several limitations to the current study. Since cancer cell line including HeLa cells used in the study is thought to be altered its phenotype, native functions and responsiveness to stimulation by genetic manipulation, our *in vitro* model may not fully represent host responses during infection of GAS. Therefore, in order to ensure that our results are not just a quirk of the particular cell line, further studies using primary cells or *in vivo* model are required for assessing the involvement of NLRX1 in the regulation of invasion and autophagy during GAS infection.

**Figure 6:**
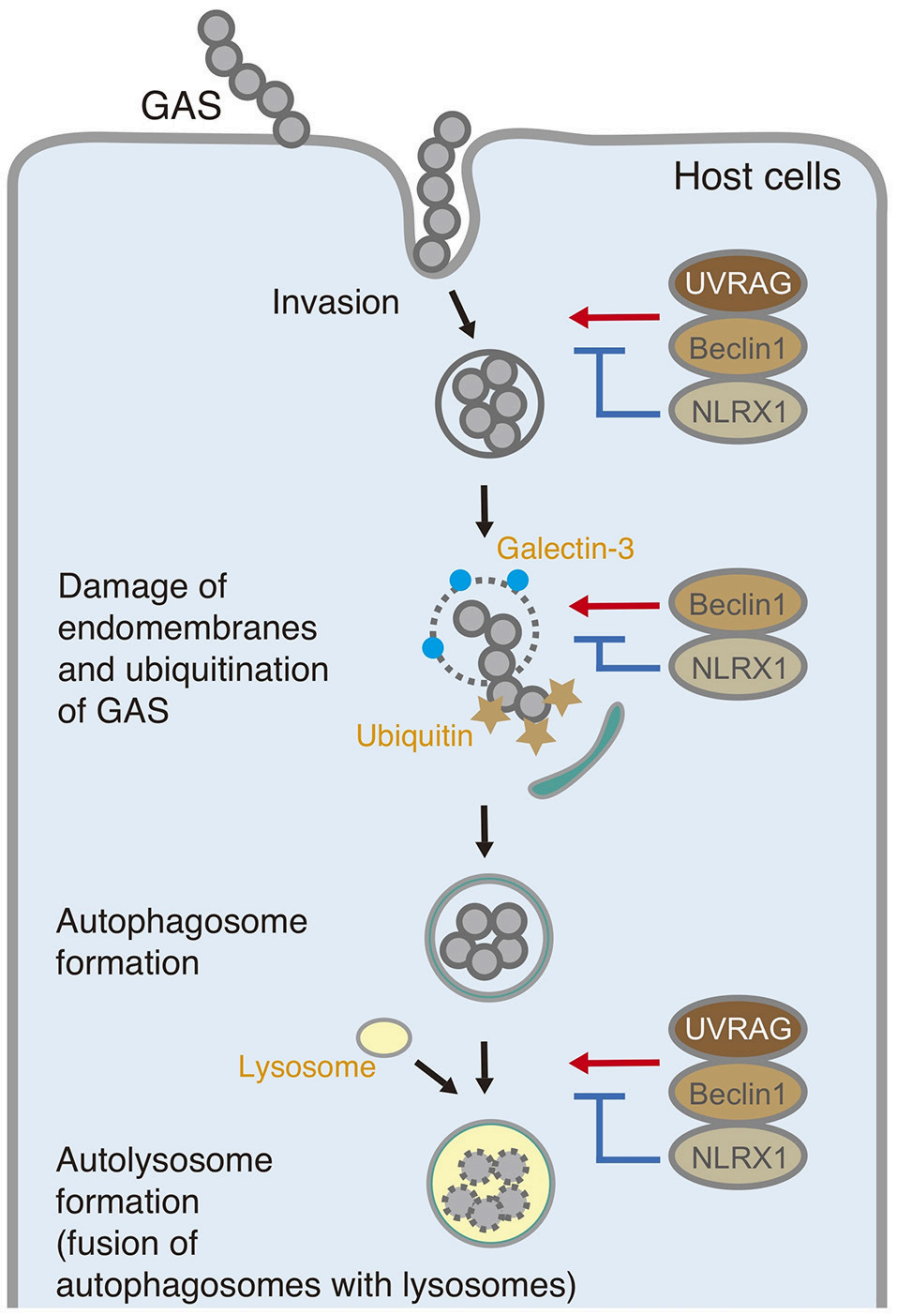
Schematic diagram of the postulated inhibitory function of NLRX1 during invasion and autophagy upon Group A *Streptococcus* (GAS) infection.

In summary, NLRX1 inhibits invasion and autophagic processes upon GAS infection by interacting with the Beclin 1–UVRAG complex (Figure 6). The data expand our understanding of the importance of NLRX1 in bacterial invasion and autophagy and could contribute to the design of further approaches to understand the fundamental aspects of invading pathogen–host cell interactions.

## Author contributions

IN, CA, and SN conceived and designed the experiments, analyzed the data, and wrote the manuscript, analyzed the data and critically revised the manuscript. CA, SN, MK, TN, AM-N, HT, and SY performed the experiments. IN and CA procured funding. All authors read and approved the final manuscript.

### Conflict of interest statement

The authors declare that the research was conducted in the absence of any commercial or financial relationships that could be construed as a potential conflict of interest.

## Supplementary material

Supplementary Figure S1 Generation of NLRX1 knockout cells using the CRISPR/Cas9 system. **(A)** Schematic illustration of *nlrx1* gene structure. The targeted locus of gRNA and the protospacer-adjacent motif (PAM) sequences were indicated by underline and red letters, respectively. **(B)** HeLa wild-type (WT) or NLRX1 knockout (KO) cells were analyzed by immunoblotting using an anti-NLRX1 antibody. **(C)** Sequences of the WT NLRX1 locus and mutated allele of the obtained NLRX1 KO cells around the target locus. Deleted nucleotides are indicated by hyphens. **(D)** PCR amplification of the target region in the deletion mutant. The primer sequences are indicated by green letters. The position of the expected PCR amplicon is indicated by an arrowhead.

Supplementary Figure S2 Generation of Beclin 1, UVRAG, and Rubicon KO cells using the CRISPR/Cas9 system and knockdown (KD) of Vps34 and Atg14. **(A–C)** HeLa wild-type and either Beclin 1 **(A)**, UVRAG **(B)**, or Rubicon **(C)** KO cells were analyzed by immunoblotting using corresponding antibodies. Sequences of the wild-type Beclin 1, UVRAG, or Rubicon locus and mutated allele of each KO cell line around the target locus. The targeted locus of gRNA and the protospacer-adjacent motif (PAM) sequences are indicated by underline and red letters, respectively. Deleted nucleotides are indicated by hyphens. **(D,E)** Immunoblotting analysis of Vps34 **(D)** and Atg14 **(E)** knockdown HeLa cells. HeLa cells were transfected with either control siRNA, or siRNA targeting Vps34 or Atg14. Expression of Vps34 and Atg14 was analyzed by western blotting using corresponding antibodies.

Supplementary Figure S3 Construction of NLRX1 deletion mutants. **(A)** Schematic representation of NLRX1 deletion mutants. **(B)** The expression profile of deletion mutants was determined by western blotting using an anti-FLAG antibody. **(C)** Confocal micrographs of HeLa cells transfected with EmGFP-tagged NLRX1 deletion mutants. Mitochondria and nuclei were stained with MitoTacker dye and DAPI, respectively. Scale bars, 10 μm.
