# Supplementary figures and images for: NLRX1 Negatively Regulates Group A Streptococcus Invasion and Autophagy Induction by Interacting With the Beclin 1–UVRAG Complex
Source: Front Cell Infect Microbiol. 2018 Nov 14;8:403. doi: 10.3389/fcimb.2018.00403 (PMC6246980; doi:10.3389/fcimb.2018.00403)

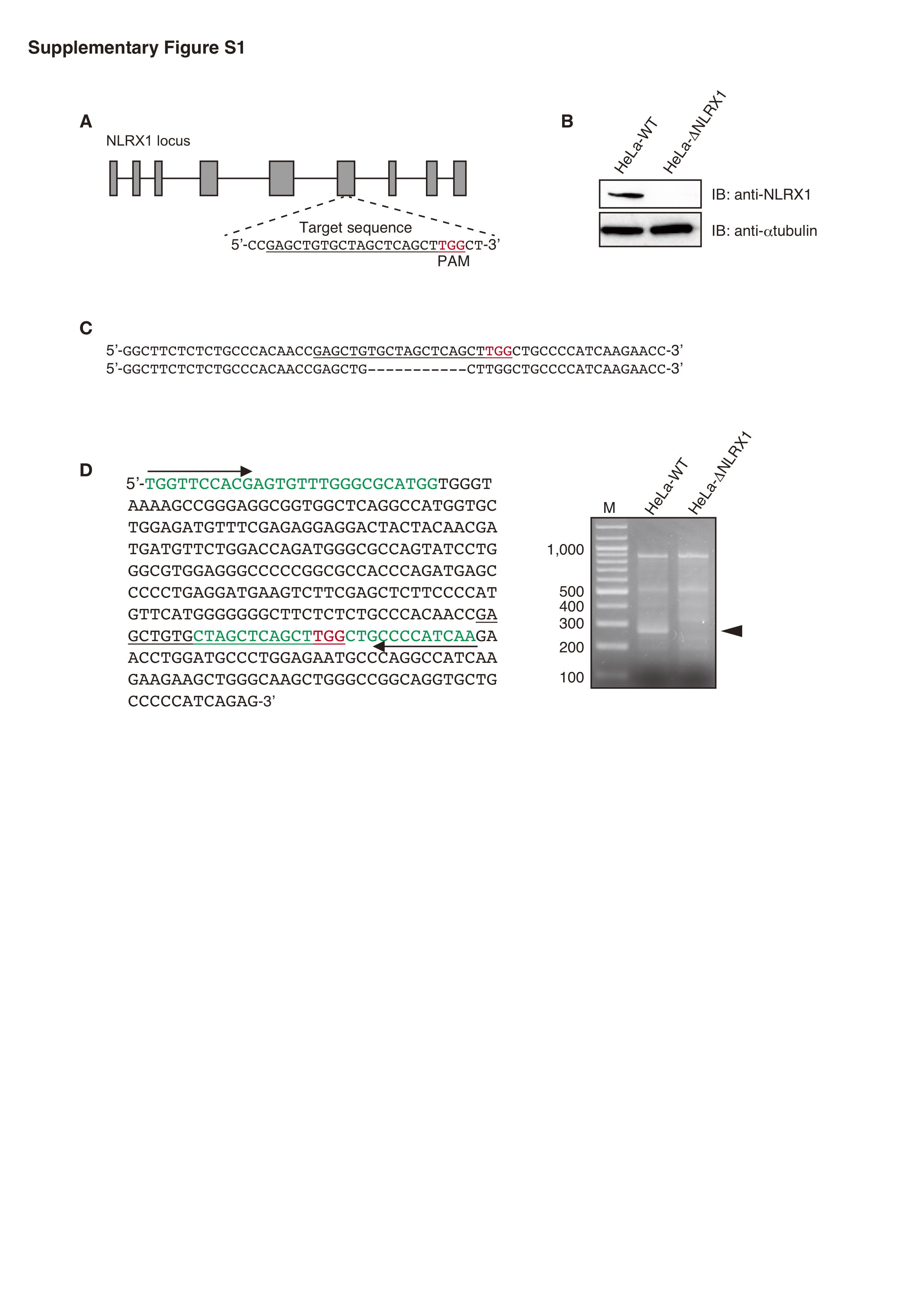

Supplement: Supplementary Figure S1 — Generation of NLRX1 knockout cells using the CRISPR/Cas9 system. (A) Schematic illustration of nlrx1 gene structure. The targeted locus of gRNA and the protospacer-adjacent motif (PAM) sequences were indicated by underline and red letters, respectively. (B) HeLa wild-type (WT) or NLRX1 knockout (KO) cells were analyzed by immunoblotting using an anti-NLRX1 antibody. (C) Sequences of the WT NLRX1 locus and mutated allele of the obtained NLRX1 KO cells around the target locus. Deleted nucleotides are indicated by hyphens. (D) PCR amplification of the target region in the deletion mutant. The primer sequences are indicated by green letters. The position of the expected PCR amplicon is indicated by an arrowhead. [file Image_1.JPEG]

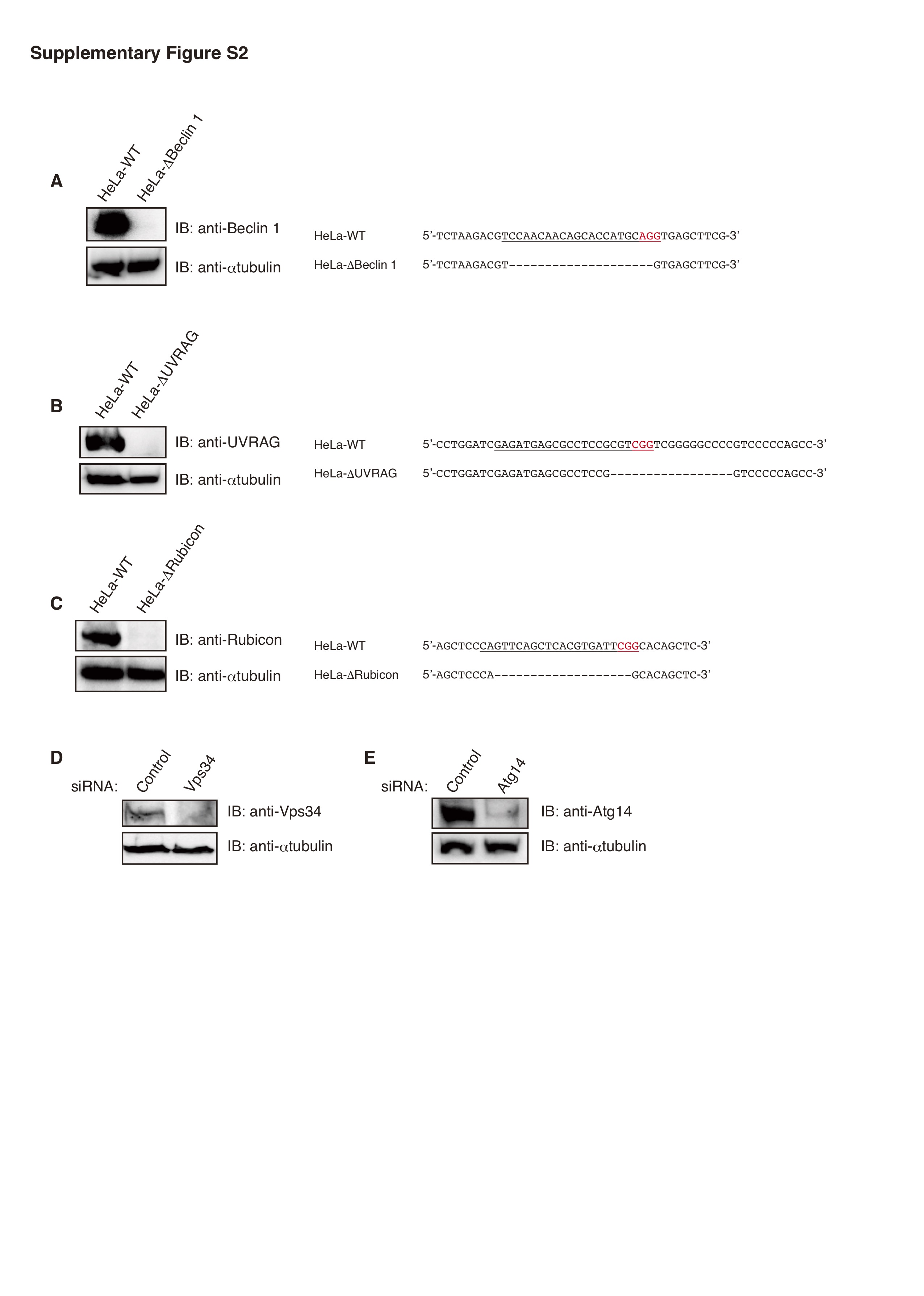

Supplement: Supplementary Figure S2 — Generation of Beclin 1, UVRAG, and Rubicon KO cells using the CRISPR/Cas9 system and knockdown (KD) of Vps34 and Atg14. (A–C) HeLa wild-type and either Beclin 1 (A), UVRAG (B), or Rubicon (C) KO cells were analyzed by immunoblotting using corresponding antibodies. Sequences of the wild-type Beclin 1, UVRAG, or Rubicon locus and mutated allele of each KO cell line around the target locus. The targeted locus of gRNA and the protospacer-adjacent motif (PAM) sequences are indicated by underline and red letters, respectively. Deleted nucleotides are indicated by hyphens. (D,E) Immunoblotting analysis of Vps34 (D) and Atg14 (E) knockdown HeLa cells. HeLa cells were transfected with either control siRNA, or siRNA targeting Vps34 or Atg14. Expression of Vps34 and Atg14 was analyzed by western blotting using corresponding antibodies. [file Image_2.JPEG]

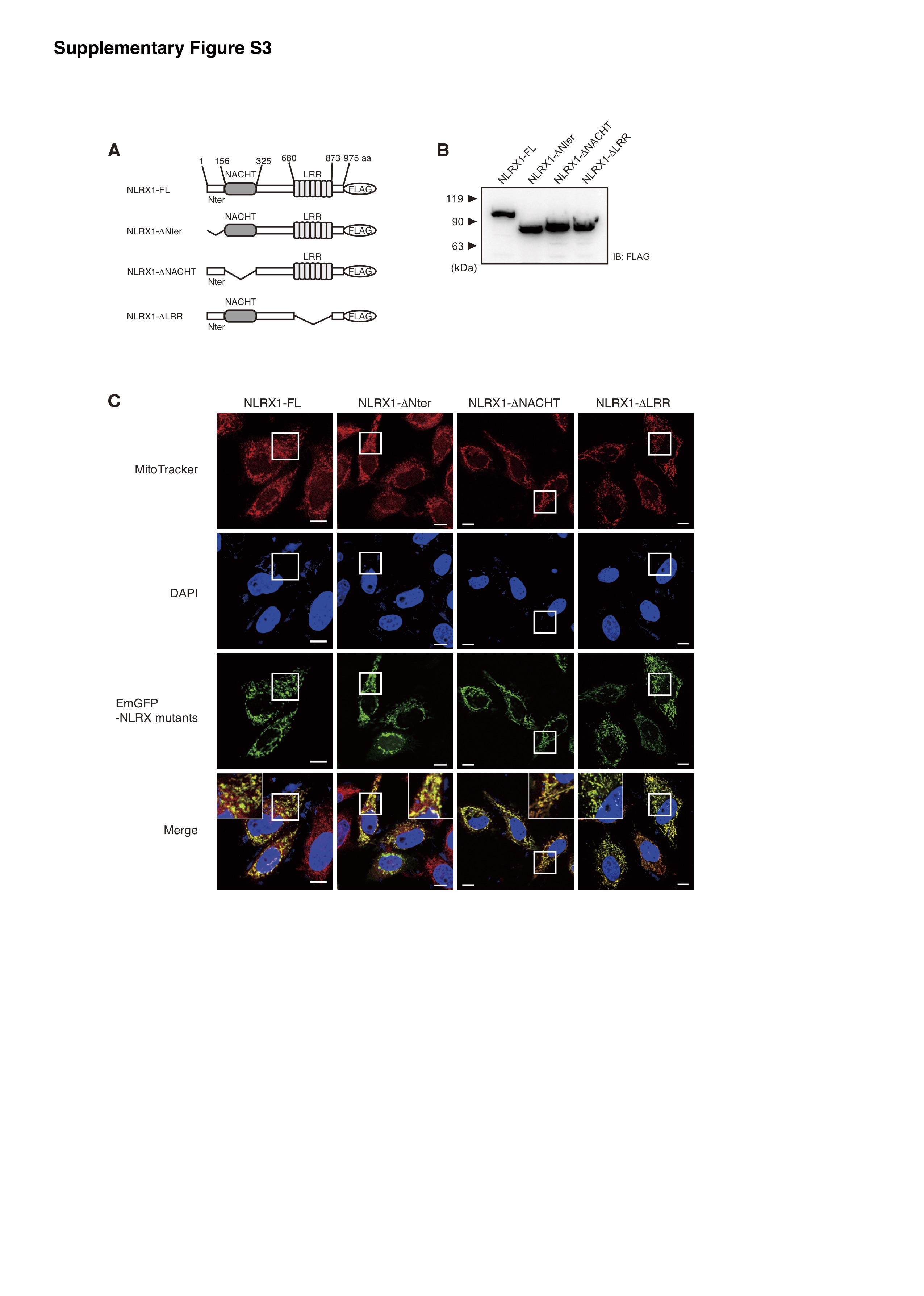

Supplement: Supplementary Figure S3 — Construction of NLRX1 deletion mutants. (A) Schematic representation of NLRX1 deletion mutants. (B) The expression profile of deletion mutants was determined by western blotting using an anti-FLAG antibody. (C) Confocal micrographs of HeLa cells transfected with EmGFP-tagged NLRX1 deletion mutants. Mitochondria and nuclei were stained with MitoTacker dye and DAPI, respectively. Scale bars, 10 μm. [file Image_3.JPEG]
